# An epidemiological analysis of potential associations between C-reactive protein, inflammation, and prostate cancer in the male US population using the 2009–2010 National Health and Nutrition Examination Survey (NHANES) data

**DOI:** 10.3389/fchem.2015.00055

**Published:** 2015-08-28

**Authors:** Catherine A. St. Hill, M. Nawal Lutfiyya

**Affiliations:** ^1^Department of Experimental and Clinical Pharmacology, College of Pharmacy, University of MinnesotaMinneapolis, MN, USA; ^2^Department of Pharmacy Practice and Pharmaceutical Sciences, College of Pharmacy, University of MinnesotaMinneapolis, MN, USA; ^3^National Center for Interprofessional Education and Practice, Children's Rehabilitation Center, University of MinnesotaMinneapolis, MN, USA

**Keywords:** prostate cancer, infection, inflammation, NHANES, C-reactive protein

## Abstract

Prostate cancer is the second leading cause of cancer-related deaths in US males, yet much remains to be learned about the role of inflammation in its etiology. We hypothesized that preexisting exposure to chronic inflammatory conditions caused by infectious agents or inflammatory diseases increase the risk of prostate cancer. Using the 2009–2010 National Health and Nutrition Examination Survey, we examined the relationships between demographic variables, inflammation, infection, circulating plasma C-reactive protein (CRP), and the risk of occurrence of prostate cancer in US men over 18 years of age. Using IBM SPSS, we performed bivariate and logistic regression analyses using high CRP values as the dependent variable and five study covariates including prostate cancer status. From 2009–2010, an estimated 5,448,373 men reported having prostate cancer of which the majority were Caucasian (70.1%) and were aged 40 years and older (62.7%). Bivariate analyses demonstrated that high CRP was not associated with an increased risk of prostate cancer. Greater odds of having prostate cancer were revealed for men that had inflammation related to disease (*OR* = 1.029, CI 1.029–1.029) and those who were not taking drugs to control inflammation (*OR* = 1.330, CI 1.324–1.336). Men who did not have inflammation resulting from non-infectious diseases had greater odds of not having prostate cancer (*OR* = 1.031, CI 1.030–1.031). Logistic regression analysis yielded that men with the highest CRP values had greater odds of having higher household incomes and lower odds of having received higher education, being aged 40 years or older, being of a race or ethnicity different from other, and of having prostate cancer. Our results show that chronic inflammation of multiple etiologies is a risk factor for prostate cancer and that CRP is not associated with this increased risk. Further research is needed to elucidate the complex interactions between inflammation and prostate cancer.

## Introduction

Prostate cancer is a slowly progressive chronic disease that is a significant health problem for aging males both in the United States (US) population and worldwide. In the US, prostate cancer is the most commonly diagnosed non-skin cancer and the second leading cause of cancer deaths. In 2013 alone, an estimated 238,590 men were diagnosed with prostate cancer and 29,720 deaths were associated with this disease (Siegel et al., 2013). Numerous epigenetic factors and somatic genetic changes promote the development of prostate cancer (Kim et al., 2007). Age (Armitage and Doll, 1954, 1957), geographic location (Goggins and Wong, 2009; Center et al., 2012), family history of the disease (Kicinski et al., 2011), and race/ethnicity (Merrill and Sloan, 2012) are well documented risk factors for prostate cancer that cause wide variation in disease incidence (Crawford, 2003; Pu et al., 2004) as well as environmental and life-style factors such as diet and obesity (Hsing and Chokkalingam, 2006). Despite this information, little is known about the precise factors leading to the development and progression of prostate cancer. A better understanding of the factors contributing to prostate cancer incidence represents an important opportunity to improve health outcomes of this disease.

Recently, epidemiologic, genetic, and molecular studies have indicated that prostate cancer may have infectious and inflammatory origins and furthermore that prostate cancer is not simply a result of aging (Sutcliffe et al., 2006; De Marzo et al., 2007; Sciarra et al., 2007; Cheng et al., 2010). Inflammation of the prostate, known as prostatitis, is a heterogeneous disease in men that may be symptomatic or asymptomatic, and is characterized by bacterial or inflammatory causes (Krieger et al., 1999; Nelson et al., 2013). Some studies have reported a positive association between prostatic inflammation and prostate cancer, although these studies had limitations such as potential detection bias and inaccuracies of self-reporting (Dennis et al., 2002; Roberts et al., 2004; Palapattu et al., 2005; Patel et al., 2005). Intriguingly, a causal link has been established for the roles of hepatitis B and hepatitis C in hepatic cancer (Alison et al., 2011), human papillomavirus virus (HPV) in cervical cancer (zur Hausen, 1996), Epstein Barr virus (EBV)—a herpes virus—in head and neck lymphomas (Burkitt, 1958), and HIV infections in an increased risk of non-Hodgkin lymphoma and Karposi's sarcoma (Goedert, 2000). These infectious agents share a common pathogenesis of long latency after viral exposure and chronic inflammation is a common component of these virally induced malignancies. A population based study found that the odds of prostate cancer were increased in men of African descent who were infected with the sexually transmitted disease gonorrhea or who developed prostatitis (Sarma et al., 2006).

C-reactive protein (CRP) is a plasma protein that rises rapidly in the circulation in response to acute inflammation, infection, and tissue damage (Gabay and Kushner, 1999; Black et al., 2004). Blood levels of CRP are also moderately elevated during chronic inflammatory diseases and cancer (Allin et al., 2009). CRP is a non-specific marker of inflammation and a positive association between elevated circulating CRP levels and risk of cancer of any type has been reported (Allin et al., 2009; Heikkilä et al., 2011). A few studies indicate that human CRP is preferentially glycosylated as a post-translational modification (Köttgen et al., 1992; Das et al., 2003). Glycosylation of CRP may differ in the carbohydrate and amino-acid sequences depending on the inducing pathological condition including infectious, non-infectious, and neoplastic diseases (Das et al., 2003). The biological function of CRP is strongly dependent on its structure and isoforms of CRP have been shown to bind to neoglycoproteins containing simple sugar structures (Lee and Lee, 2006). Glycosylation of human CRP and its carbohydrate binding properties under different pathological conditions may serve as a marker of disease and influence its biological function in prostate cancer. It is therefore important to further investigate the relationship between CRP and prostate cancer. Prostatitis was reported to be a precursor to prostate cancer but elevated circulating levels of CRP were not predominantly associated with increased risk of prostate cancer (Platz et al., 2004; Siemes et al., 2006; De Marzo et al., 2007; Allin et al., 2009; Heikkilä et al., 2011). In contrast, recent studies have shown that CRP participates in tumor proliferation and high serum concentrations of CRP were correlated with shorter overall survival in patients with castration-refractory prostate cancers (Beer et al., 2008).

Given this body of evidence, it is plausible to infer that chronic inflammation resulting from infections or from other causes may predispose affected men to develop prostate cancer however a need exists to further examine these relationships. Our study sought to more clearly define the associations between the occurrence of infection and inflammation of varied etiologies, and the incidence of prostate cancer using a large population based cohort of males in the US. We examined CRP as an inflammatory indicator of the risk of prostate cancer. We also investigated the role of non-steroidal anti-inflammatory drugs (NSAIDS) in these relationships since regular NSAID use is associated with a modestly reduced risk of prostate cancer (Nelson and Harris, 2000; Jacobs et al., 2005). In this study, we hypothesized that preexisting exposure to chronic inflammatory conditions caused by infectious agents or inflammatory diseases increase the risk of occurrence of prostate cancer.

## Materials and methods

To answer the research question, 2009–2010 NHANES data were analyzed. The NHANES is conducted by the Centers for Disease Control and Prevention's (CDC)^1^ National Center for Health Statistics (NCHS) and is NCHS' most in-depth and logistically complex survey. NHANES collects nationally representative data on the health and nutritional status of the non-institutionalized, civilian population of the US. To collect these data the survey employs a stratified and multistage probability sampling design. There are two parts to the NHANES. The first part entails collecting data using a standardized personal/household interview protocol. For the second part of NHANES, standardized physical examinations, diagnostic procedures, and laboratory tests of biologic samples are undertaken at mobile examination trailers. These dual data collection techniques and efforts are used to obtain information about diagnosed and undiagnosed conditions; growth and development, including overweight and obesity; diet and nutrition; risk factors; and environmental exposures. While NHANES data have been collected since the early 1960s, in 1999, the survey became a continuous yet nimble endeavor allowing for changing foci on a variety of health and nutrition measurements to meet emerging needs.

To ensure national representativeness, the NCHS and CDC recommend that all analyses of NHANES data be conducted on weighted data. The weighting of sample data permits analysts to produce estimates of the statistics that would have been obtained if the entire sampling frame had been surveyed. Sample weights can be considered as measures of the number of persons represented by the particular sampled participant. A more detailed explanation of the survey design for the NHANES, including approval from the CDC institutional review board for data collection and analysis, is available elsewhere^1^.

Four demographic variables were included in the analyses performed. They were race/ethnicity, age, education, and annual household income. For analysis purposes, these variables were all recoded from their original format. Recoding the variables entailed reducing categories (race/ethnicity, household income, and education) or transforming the variable from a continuous to a categorical one (age). For all of the demographic variables the categories of “don't know” and “refused” were removed.

Four inflammation proxy variables were used in the analyses performed. These were: circulating plasma CRP (high grade inflammation was defined as CRP values >3 mg/ml and low grade inflammation as CRP values of ≤3 mg/ml); inflammation due to infection; drug use to control inflammation; and inflammation resulting from existing disease. The last three of these four variables were computed from multiple variables as indicated in the Chart 1 below.

**CHART 1:** **Computed variables with contributing variables, NHANES 2009–2010**.

**Computed variable**	**Contributing variable**
Inflammation from infection	Positive result for Epstein Barr virus
	Positive result for HIV antibody
	Positive result for Hepatitis B core antibody
	Positive result for Hepatitis B surface antigen
	Positive result for Hepatitis C antibody (confirmed)
	Positive result for Hepatitis C RNA (HCV-RNA)
	Positive result for Herpes Simplex Virus I
	Positive result for Herpes Simplex Virus II
Inflammation indicated from drugs	Take prednisone or cortisone daily
	Taken ibuprofen for pain
	Taken naproxyn for pain
	Taken indomethacin for pain
	Taken cox-2 inhibitor for pain
	Taken aspirin for pain
Inflammation indicated from disease	Have osteoporosis/brittle bones
	Have diabetes
	Have arthritis
	Have congestive heart failure
	Have coronary heart disease
	Have angina/angina pectoris
	Have had a heart attack

Two bivariate analyses were performed. The first to describe the study population by prostate cancer status (have prostate cancer vs. did not have prostate cancer); the second, to examine the unadjusted relationship between the dependent variable (Prostate Cancer Status) by the computed inflammation covariates. One logistic regression model was tested with High Cancer Risk C-Reactive Protein Value as the dependent variable with five study covariates entered into the model. In the logistic regression analysis, prostate cancer status was entered into the model as a covariate.

Alpha was set at 0.05 for all tests of statistical significance. SPSS version 22.0 (IBM, Chicago, IL) was used to complete the analyses. The Institutional Review Board (IRB) at the researchers' institution recognize that the analysis of de-identified and publicly available data does not constitute human subjects research as defined in federal regulations and as such does not require IRB review. Hence, human subjects' approval was not necessary nor sought since this was a de-identified data only study.

## Results

Table 1 displays the bivariate analysis result of five of the study covariates by the dependent variable prostate cancer status. The population included in this analysis was men 18 years of age and older for the 2009–2010 NHANES survey. An estimated 296,495,347 men reported having no prostate cancer while 5,448,373 reported having prostate cancer. Of those men with prostate cancer, 70.1% were Caucasian, 13.5% were African American or Black, 13.4% were Hispanic, and 3.1% reported their race to be “Other” including multiracial status. The majority of men aged 40 years and older (62.7%) reported having prostate cancer. While all the differences between men with and without prostate cancer were statistically significant, this is not surprising since the analyses were performed on large sample weighted data.

**Table 1:** **Description of study population by prostate cancer status, 2009–2010 NHANES data**.

**Variables and factors**		**% Prostate cancer status**		***P* (for *z*-test)**
		No prostate cancer (*n* = 296495347)^*^	Has prostate cancer (*n* = 5448373)^*^	
Race/Ethnicity	Hispanic	16.0	13.4	< 0.05
	Caucasian	64.5	70.1	< 0.05
	African American or Black	12.0	13.5	< 0.05
	Other	7.5	3.1	< 0.05
Age	18–39 years	39.1	37.3	< 0.05
	40 and older	60.9	62.7	< 0.05
Education	< HS	18.9	26.4	< 0.05
	HS graduate	53.4	48.3	< 0.05
	University graduate	27.7	25.3	< 0.05
Annual household income	< $54,999	48.1	52.8	< 0.05
	≥$55,000–99,999	29.7	23.1	< 0.05
	$100,000 and over	22.1	24.1	< 0.05
C-reactive protein	Low risk for cancer	43.1	39.9	< 0.05
	No cancer risk	28.6	32.9	< 0.05
	High risk for cancer	28.4	27.2	< 0.05

* *Weighted; HS, High School*.

Table 2 displays the study's four inflammation related variables by prostate cancer status. For CRP, men with a low risk of cancer by CRP measure had slightly higher odds of having prostate cancer. When examining the relationship between inflammation from infection and prostate cancer status, men that did not have diseases such as EBV, HIV, hepatitis B and C, and HSV I and II that are associated with inflammation had slightly greater odds of not having prostate cancer. The analysis of men taking anti-inflammatory drugs for a presumed inflammatory condition by prostate cancer status revealed that men that were not taking anti-inflammatory drugs had greater odds of having prostate cancer. Finally, the computed variable of inflammation indicated from inflammatory diseases by prostate cancer status indicated that men without disease-related inflammation had greater odds of not having prostate cancer.

**Table 2:** **Inflammation covariates by prostate cancer status, 2009–2010 NHANES data**.

**Variables and factors**		**Percent by prostate cancer status**		**Unadjusted odds ratios (%95 CI)**
		**No prostate cancer**	**Has prostate cancer**	
C-reactive protein	Low grade inflammation (≤3 mg/ml)	71.6	72.8	*OR* = 1.061 (1.059–1.064) Men with low grade inflammation by CRP measure had slightly higher odds of having prostate cancer
	High grade inflammation (>3 mg/ml)	28.4	27.2	
Inflammation from infection	No inflammation from infection	68.9	35.0	*OR* = 1.029 (1.029–1.029) Men with no inflammation from infection had slightly greater odds of not having prostate cancer
	Inflammation from infection	31.1	65.0	
Drug use to control inflammation	No drugs taken for inflammation	14.6	18.7	*OR* = 1.330 (1.324–1.336) Men who were not taking drugs to control inflammation had greater odds of having prostate cancer
	Drugs taken for inflammation	85.4	81.3	
Existing disease causing inflammation	No disease causing inflammation	63.4	54.0	*OR* = 1.031 (1.030–1.031) Men with no inflammation from disease had greater odds of not having prostate cancer
	Disease causing inflammation	36.6	46.0	

For the logistic regression analysis performed, prostate cancer was entered into the model as a covariate along with race/ethnicity, annual household income, education, and age (Table 3). The category of high CRP values was the dependent variable in this model. Analysis yielded that men with the highest CRP values had greater odds of having higher household incomes. These men with high CRP values had lower odds of having received higher education, being aged 40 years or older, being of a race or ethnicity different from other, and of having prostate cancer.

**Table 3:** **Logistic regression with high grade (>3 mg/ml) inflammation C-reactive protein values as the dependent variable 2008–2009 NHANES data**.

**Variables**	**Factors**	**Adjusted odds ratios (95% CI)**
Race/Ethnicity	Hispanic	0.818 (0.817, 0.819)
	Caucasian	0.961 (0.960, 0.963)
	African American or Black	0.906 (0.904, 0.907)
	Other	^*^–
Annual household income	< $54,999	^*^–
	≥$55,000–99,999	1.106 (1.105, 1.107)
	$100,000 and over	1.122 (1.121, 1.123)
Education	< HS	^*^–
	HS graduate	0.761 (0.761, 0.762)
	University graduate	0.804 (0.803, 0.805)
Age	18–39 years	^*^–
	40 and older	0.919 (0.919, 0.920)
Prostate cancer	No prostate cancer	^*^–
	Prostate cancer	0.854 (0.852, 0.856)

## Discussion

Evidence from histopathologic, molecular, and epidemiologic studies indicates that prostatic inflammation plays a vital role in the development of prostate cancer (Nelson et al., 2004; Pollard, 2004; Balkwill et al., 2005; De Marzo et al., 2007). Several studies on the association between levels of the inflammatory marker CRP and risk of prostate cancer have reported negative results however these studies used small sample sizes in case-controlled study designs (Platz et al., 2004; Siemes et al., 2006; De Marzo et al., 2007; Allin et al., 2009; Heikkilä et al., 2011). Using 2009–2010 NHANES data, we sought to study the potential associations between inflammation and the occurrence of prostate cancer further by using the inflammatory marker CRP and by employing a large sample size of over 5 million men of different races and economic backgrounds who were 18 years of age or older.

In agreement with the previously mentioned reports, we found that CRP was not a reliable indicator of risk of prostate cancer as men with CRP values ≤3 mg/ml (low levels of inflammation) had slightly higher odds of having prostate cancer. In support of our hypothesis that inflammation related to infection and other causes increased the risk of occurrence of prostate cancer, we observed that infection with EBV, HIV, Hepatitis B or C, or HSV I or II and the corresponding inflammation associated with these diseases increased the risk of occurrence of prostate cancer. Similarly, those men who reported having inflammatory diseases such as osteoporosis, diabetes mellitus, arthritis, or cardiovascular diseases were at a greater risk of having prostate cancer. Several epidemiological studies have found an increased risk of prostate cancer in association with a history of prostatitis (Dennis et al., 2002; Roberts et al., 2004; Patel et al., 2005) or infection with any sexually transmitted diseases (STD) including syphilis, gonorrhea, and human papilloma virus (Dennis and Dawson, 2002; Patel et al., 2005). Limitations of many of these studies were the inclusion of primarily Caucasian men or populations in which the prevalence of prostate cancer and/or STDs was low, or small sample sizes. We add novel information to existing knowledge by using a large population-based sample size to demonstrate that chronic inflammation from multiple etiologies including some viral infections (sexually and non-sexually transmitted) and the existence of non-infectious inflammatory diseases are risk factors for prostate cancer.

We observed that men who were taking NSAIDS were at a lower risk of having prostate cancer. Several epidemiological studies examining the association between the use of NSAIDs and the risk of prostate cancer suggest an inverse relationship however reports are inconsistent (Nakai and Nonomura, 2013). These discrepancies may be due to differences in doses and duration of treatment, and screening bias by healthcare providers. In our study, NSAID use was self-reported and we included men with any previous history of taking NSAIDS.

Age (Armitage and Doll, 1954, 1957), geographic location (Goggins and Wong, 2009; Center et al., 2012), family history of the disease (Kicinski et al., 2011), and race/ethnicity (Merrill and Sloan, 2012) are well-established risk factors for prostate cancer. In our study, we performed logistic regression analysis using high levels of CRP (>3 mg/ml indicating increased risk of any type of cancer) as the dependent variable. We found that high CRP values indicating high inflammation were not positively correlated with the occurrence of prostate cancer. Other variables in the logistic regression model (race/ethnicity, annual household income, education, and age) were used to control for confounding effects between the variables of high CRP values (the dependent variable) and prostate cancer (the independent variable). Our finding that men with the highest CRP values indicating highest cancer risk had greater odds of having higher household incomes is in agreement with some studies (Rimpela and Pukkala, 1987; Yu et al., 1988), but inconsistent with others (Oishi et al., 1989; Fincham et al., 1990) and a few studies have reported no association (Talamini et al., 1986; Severson et al., 1989). Possible reasons for our finding are that men with higher household incomes may have greater medical attention, easier access to higher quality healthcare, and different attitudes and concern over health matters than men with lower household incomes.

The limitations of our study were mostly attributable to how the survey data were collected. First, the NHANES database is a cross-sectional survey of the U.S. population and therefore does not have longitudinal relationships with participants. Since the information is from self-reported data, we may not have captured all actual cases of prostate cancer. Second, we could only examine covariates that were available from the 2009–2010 NHANES because the data pertaining to our study was not consistently available and reported for all years. For this reason, we could not estimate the prevalence of prostate cancer. Third, we were not able to obtain information on mortality and the geographic distribution of respondents as this information is not collected from NHANES.

Despite these limitations, our study had strengths worth noting. Since we used national patient record population-level survey data, we had a large data set that was weighted to ensure that our findings could be more easily and accurately generalized to the US population. Our pilot study demonstrated that NHANES can be used to examine relationships between CRP, demographic variables, inflammation, and prostate cancer. Chronic inflammation resulting from infectious or non-infectious diseases can increase the risk of having prostate cancer. Men with a higher household income are at a higher risk of having prostate cancer. Future studies will be needed in order to elucidate the complex relationships between inflammation and prostate cancer to be able to develop novel strategies to target the inflammatory process and combat this malignancy.

## Author contributions

CSH conceived the work. CSH and ML equally substantially contributed to the design of the work and to the acquisition, analysis, and interpretation of data for the work; drafted the work; revised it critically for important intellectual content; and approved the version to be submitted. The both agree to be accountable for all aspects of the work in ensuring that questions related to the accuracy or integrity of any part of the work are appropriately investigated and resolved. This work was supported by internal funds provided by the College of Pharmacy, University of Minnesota.

### Conflict of interest statement

The authors declare that the research was conducted in the absence of any commercial or financial relationships that could be construed as a potential conflict of interest.

## Footnotes

^1^Centers for Disease Control and Prevention. About the National Health and Nutrition Examination Survey (NHANES) available at www.cdc.gov/nchs/about/major/nhanes/intro_mec.htm. Accessed on May 13, 2013.
